# Therapeutic effects of peripherally administrated neural crest stem cells on pain and spinal cord changes after sciatic nerve transection

**DOI:** 10.1186/s13287-021-02200-4

**Published:** 2021-03-15

**Authors:** Yang Zhang, Xiang Xu, Yuxin Tong, Xijie Zhou, Jian Du, In Young Choi, Shouwei Yue, Gabsang Lee, Blake N. Johnson, Xiaofeng Jia

**Affiliations:** 1 Department of Physical Medicine & Rehabilitation, Qilu Hospital, Cheeloo College of Medicine, Shandong University, Jinan, 250012 Shandong China; 2 Department of Neurosurgery, University of Maryland School of Medicine, 685 West Baltimore Street, MSTF Building 823, Baltimore, MD 21201 USA; 3 Department of Industrial and Systems Engineering, School of Neuroscience, Virginia Tech, Blacksburg, 24061 VA USA; 4 Department of Neurology, Johns Hopkins University School of Medicine, Baltimore, MD 21205 USA; 5 Department of Neuroscience, Johns Hopkins University School of Medicine, Baltimore, MD 21205 USA; 6 Department of Orthopedics, University of Maryland School of Medicine, Baltimore, MD 21201 USA; 7 Department of Anatomy and Neurobiology, University of Maryland School of Medicine, Baltimore, MD 21201 USA; 8 Department of Biomedical Engineering, Johns Hopkins University School of Medicine, Baltimore, MD 21205 USA; 9 Department of Anesthesiology and Critical Care Medicine, Johns Hopkins University School of Medicine, Baltimore, MD 21205 USA

**Keywords:** Peripheral nerve injury, Spinal cord, Neuropathic pain, Neural crest stem cells, Glial activation

## Abstract

**Background:**

Severe peripheral nerve injury significantly affects patients’ quality of life and induces neuropathic pain. Neural crest stem cells (NCSCs) exhibit several attractive characteristics for cell-based therapies following peripheral nerve injury. Here, we investigate the therapeutic effect of NCSC therapy and associated changes in the spinal cord in a sciatic nerve transection (SNT) model.

**Methods:**

Complex sciatic nerve gap injuries in rats were repaired with cell-free and cell-laden nerve scaffolds for 12 weeks (scaffold and NCSC groups, respectively). Catwalk gait analysis was used to assess the motor function recovery. The mechanical withdrawal threshold and thermal withdrawal latency were used to assess the development of neuropathic pain. Activation of glial cells was examined by immunofluorescence analyses. Spinal levels of extracellular signal-regulated kinase (ERK), NF-κB P65, brain-derived neurotrophic factor (BDNF), growth-associated protein (GAP)-43, calcitonin gene-related peptide (CGRP), and inflammation factors were calculated by western blot analysis.

**Results:**

Catwalk gait analysis showed that animals in the NCSC group exhibited a higher stand index and Max intensity At (%) relative to those that received the cell-free scaffold (scaffold group) (*p* < 0.05). The mechanical and thermal allodynia in the medial-plantar surface of the ipsilateral hind paw were significantly relieved in the NCSC group. Sunitinib (SNT)-induced upregulation of glial fibrillary acidic protein (GFAP) (astrocyte) and ionized calcium-binding adaptor molecule 1 (Iba-1) (microglia) in the ipsilateral L4–5 dorsal and ventral horn relative to the contralateral side. Immunofluorescence analyses revealed decreased astrocyte and microglia activation. Activation of ERK and NF-κB signals and expression of transient receptor potential vanilloid 1 (TRPV1) expression were downregulated.

**Conclusion:**

NCSC-laden nerve scaffolds mitigated SNT-induced neuropathic pain and improved motor function recovery after sciatic nerve repair. NCSCs also protected the spinal cord from SNT-induced glial activation and central sensitization.

**Supplementary Information:**

The online version contains supplementary material available at 10.1186/s13287-021-02200-4.

Treatment of peripheral nerve injury (PNI) remains a worldwide clinical challenge. Severe PNI significantly affects patient quality of life and causes significant socioeconomic burden [1–3]. While autologous nerve grafting remains the gold standard for PNI repair, it has several limitations [1, 4]. Tissue-engineered nerve grafts, such as nerve guidance conduits, have emerged as a potential alternative for autologous nerve grafts [5]. For example, we recently utilized 3D printing to fabricate anatomical nerve guidance conduits with gradients of growth factors (nerve growth factor and glial cell derived neurotrophic factor) that contained physical (topographical) and biochemical cues to promote the regeneration of a sciatic nerve tibial bifurcation injury model [6]. While it is well established that topographical and biochemical cues can assist the regeneration of peripheral nerve, the therapeutic efficacy of cells within scaffolds remains an area of active investigation [7].

It has been demonstrated that PNI changes both primary afferent and second-order spinal cord neurons, leading to sensitization and abnormal responses to peripheral stimuli [8]. Chronic patient deficits are associated with dysregulation of brain and spinal cord circuitry following PNI [9, 10]. Following injury, the spinal cord has been shown to adapt in a functionally meaningful way in number and efficacy of synaptic connections, which is referred to as spinal plasticity [11]. Protective therapies can enhance peripheral nerve regeneration, minimize secondary injury, and accelerate motor or sensory functional recovery [12]. Intrathecally or locally injected stem cells have the potential to enter the host spinal cord [12–14] and counteract the degeneration of dorsal horn neurons, thereby relieving pain [15] and enhancing peripheral nerve regeneration. Collagen scaffolds with human umbilical cord mesenchymal stem cells (MSCs) transplanted into the injury site of a patient with complete spinal cord injury resulted in the recovery of the sensory and motor functions [16]. The combination of cell transplantation and nerve transfer strategies has been shown to benefit the distal stump microenvironment of damaged nerve [17]. However, the regenerative potential of stem cells within the spinal cord and subsequent peripheral nerve recovery have not been fully addressed. For example, the location of stem cell transplantation is often near the spine. Few studies have observed the protective effect of peripheral stem cell application on the spinal cord after PNI. Hence, investigating the influence of stem cells locally administered to an injured peripheral nerve on changes in the spinal cord could lead to potential safe and effective alternative treatments that achieve improved functional clinical outcomes.

Poor response to common PNI therapies can unfortunately cause neuropathic pain and the development of central sensitization [8, 18, 19]. Although the mechanism underlying the alleviation of pain behavior is not yet fully understood, stem cells have been shown to regulate pain behavior [20, 21]. Our previous study found that intrathecal injection of bone marrow mesenchymal stem cells (BMSCs) was effective in pain relief [21]. Among various types of stem cells that were investigated, neural crest stem cells (NCSC) exhibit several characteristics that make them particularly suitable for PNI therapy [22]. We previously showed that with optimized electrical stimulation, NCSCs significantly improve peripheral nerve repair in a 15-mm sciatic nerve injury model [23]. However, it is not clear whether there is a therapeutic effect of NCSCs on central sensitization in the spinal cord that leads to pain relief and improved outcomes after sciatic nerve transection (SNT). While intraspinal injection of BMSCs was reported to suppress the NF-κB and p-p38 MAPK pathways in the spinal cord after spinal cord injury (SCI) [24], it is still unknown whether the ERK and NF-κB signals, which are associated with spinal glial activation and central sensitization [25–27], are associated with the effect of peripherally applied NCSCs. The relevant underlying mechanisms remain unelucidated.

Our objective was to investigate the therapeutic effect of scaffold-based NCSC transplantation to the sciatic nerve after SNT on the spinal cord. We hypothesize that NCSC introduction to injured peripheral nerve after SNT can relieve neuropathic pain and protect the spinal cord by downregulation of ERK and NF-κB signals and suppression of glia cells.

## Materials and methods

### Sciatic nerve transection and repair model

The IACUC of the University of Maryland School of Medicine reviewed and approved the experimental protocol. Fourteen athymic nude rats (200–250 g) were randomly divided into cell-free and cell-laden scaffold groups (7 rats per group), which are referred to as the scaffold and NCSC groups, respectively. Under inhalation of isoflurane, a previously utilized SNT and repair model was employed [6]. For the NCSC group, 2 × 10^6^ NCSCs were suspended in a 15-μl mixture of growth medium and subsequently injected to the conduit as we previously described [23].

Before and after the experiments, the animals had free access to food and water and were subjected to a 12-h day/night cycle in a quiet environment. After the behavioral test was performed 12 weeks following the operation, the rats were sacrificed under deep anesthesia, and the L4 and L5 lumbar vertebrae were harvested for subsequent studies.

### Behavioral testing

An automated gait analysis system (CatWalk XT; Noldus) was used for functional analysis of the locomotor pattern. Each animal performed at least three successful runs. All four paws were automatically marked by the software and inspected manually by a blinded experimenter as we previously described [6, 23, 28]. The parameters of stand index (speed at which the paw loses contact with the glass plate) and Max intensity At (%) (time in seconds since the start of the run that the maximum intensity is measured) were assessed.

Mechanical withdrawal threshold (MWT) was determined by the same experimenter at 12 weeks after SNT induction using the BEM-404 mechanical analgesia tester [Chinese Academy of Medical Sciences (CAMS), Beijing, China] [21]—the cutoff force was set at 50 g. A rigid tip was applied perpendicularly to the medial-plantar surface of the hind paw [29]. Brisk withdrawal or paw flinching was considered a positive response. Three successive stimuli were applied. The mean of three successive measurements was recorded as the MWT of each animal.

Thermal withdrawal latency (TWL) was measured using the Ugo Basile thermal plantar (No.37370-001, Gemonio VA Italy) as we previously described [21, 30]. The thermal intensity was set at 45 °C. Radiant light (5-mm separation distance) was focused on the medial-plantar of the hind paw. TWL was recorded when the rat lifted or licked the hind paw. A 25-s cutoff period was used to avoid tissue damage. The test was repeated three times for each rat with a 5-min interval between successive measurements. The mean of three successive measurements was recorded as the TWL for each animal.

The changes of MWT, TWL, stand index, and Max intensity At (%) were calculated using the ratio of the right hind (RH) limb (contralateral) to the left hind (LH) limb (ipsilateral).

### Western blotting

Tissue samples of the dorsal region of the spinal cord (L4–5, semi-sectioning along the longitudinal-horizontal direction) were removed. Equal amounts of the protein were separated using sodium dodecyl sulfate-polyacrylamide gel electrophoresis (SDS-PAGE) and transferred to a polyvinylidene fluoride membrane (PVDF). Primary antibodies against the following proteins were used: brain-derived neurotrophic factor (BDNF, 1:100, SC-20981, Santa Cruz), c-fos (1:500, SC-52, Santa Cruz), growth-associated protein 43 (GAP43, 1:500, ab121217, Abcam), p-ERK (1:500, SC-7383, Santa Cruz), ERK 1/2 (1:500, SC-514302, Santa Cruz), transient receptor potential vanilloid 1 (TRPV1, 1:500, ab203103, Abcam), and inducible nitric oxide synthase (i-NOS, 1:500, ab3523, Abcam). Target protein expression was normalized to β-actin (1:2000, 8457S, Cell Signaling Technology) expression. The quantification of band intensity was carried out using Image J (National Institutes of Health, USA).

### Immunofluorescence analysis

Following anesthetization, rats were perfused transcardially with 4% paraformaldehyde (PFA) and the L4–5 spinal cord was harvested. Standard immunohistochemistry procedures as we previously described were employed [23]. Briefly, the spinal cord sections were first incubated with the primary antibodies against ionized calcium-binding adapter molecule 1 (Iba-1, 1:200, Ab107159, Abcam), glial fibrillary acidic protein (GFAP, 1:200, 180063, Invitrogen), and calcitonin gene-related peptide (CGRP, 1:200, ab81887, Abcam) for 24 h at 4 °C. The specimens were then washed in PBS and incubated in the secondary antiserum solution. Antigens were observed using the following secondary antibodies: Invitrogen Alexa Fluor-488 donkey anti-goat secondary antibody, Invitrogen Alexa Fluor 488-conjugated goat anti-rabbit, and Abcam Alexa Fluor 488-conjugated goat anti-mouse (diluted 1: 500; USA) 2 h at 37 °C. Auto-fluorescence that occurred upon incubation of the tissue samples in secondary antibody solutions in the absence of the primary antibody served as a negative control.

Following washing, the tissue sections were covered and mounted with ProLong™ Gold Antifade Mountant with DAPI (4′,6-diamino-2-phenylindole, blue, Invitrogen). Fluorescence micrographs were then obtained (DMi8 microscope; Leica Microsystems). Each datum is the result of studies from four animals and five sections per animal obtained in a blinded fashion. For each image, a threshold for positive staining was determined that included all cell bodies, while excluding background staining, using Image J (National Institutes of Health, USA), as described in our previous work [23, 31]. The positive labeling in each region was expressed as percentage area and averaged intensity of staining across the dorsal and ventral horn in five randomized microscopic fields in each section for all representative images [32].

### Statistical analysis

All data are presented as mean ± standard error of the mean (SEM). The one-way analysis of variance (ANOVA) test was used for the statistical analysis of the scaffold and NCSC groups using SPSS 22.0 software (IBM Company, USA). A *p* value < 0.05 was considered statistically significant.

## Results

### NCSC therapy promotes locomotor function and attenuates sciatic nerve transection-induced pain

**Fig. 1:**
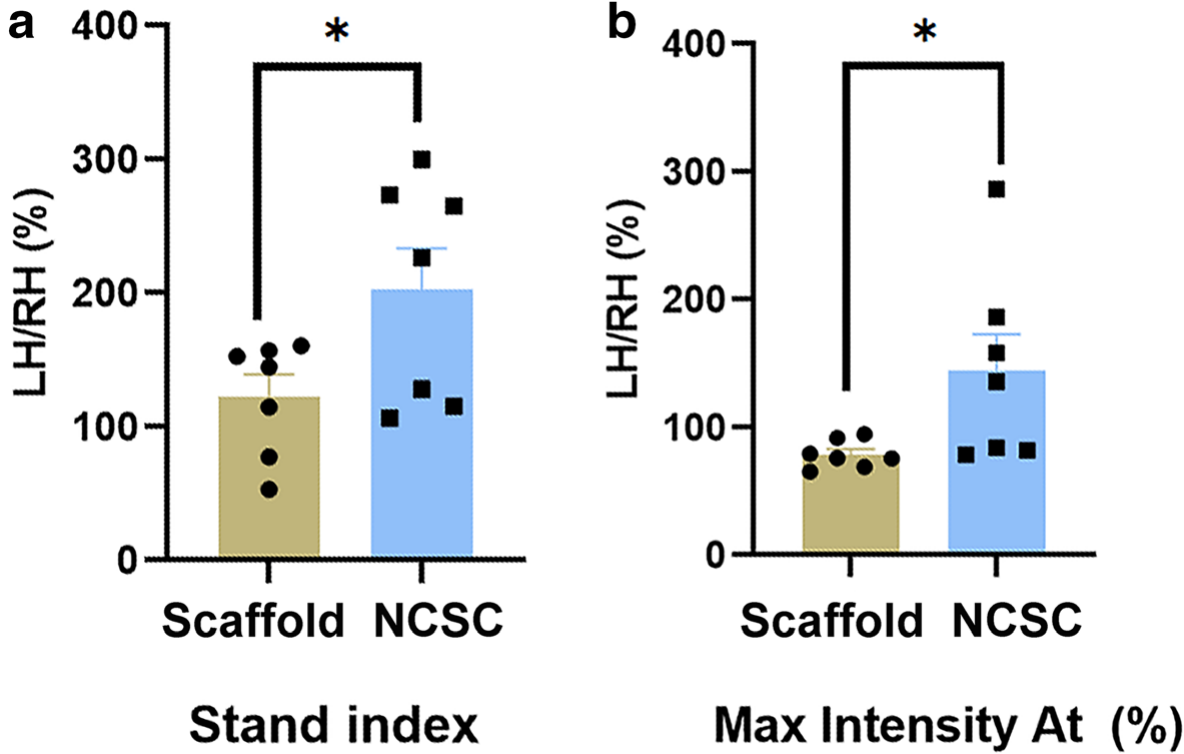
Behavioral testing for SNT-induced locomotion impairment. Catwalk gait analysis with data expressed as the ratio of values for the lesioned hind leg (left) to the contralateral hind leg (right). The NCSCs further promoted locomotor recovery in Stand index (**a**) and Max Intensity At (%) (**b**). *p < 0.05, *N* = 7

The LH/RH ratios of the stand index in the NCSC group (202.24 ± 31.41) were significantly larger than the scaffold group (122.79 ± 16.10, *p* < 0.05, Fig. 1a). The LH/RH ratio of the Max intensity At (%) in the NCSC group (144.55 ± 28.43) was also significantly larger than the scaffold group (78.97 ± 4.11, *p* < 0.05, Fig. 1b), suggesting that scaffold-based NCSC administration to peripheral nerve following SNT promoted locomotor recovery.

**Fig. 2:**
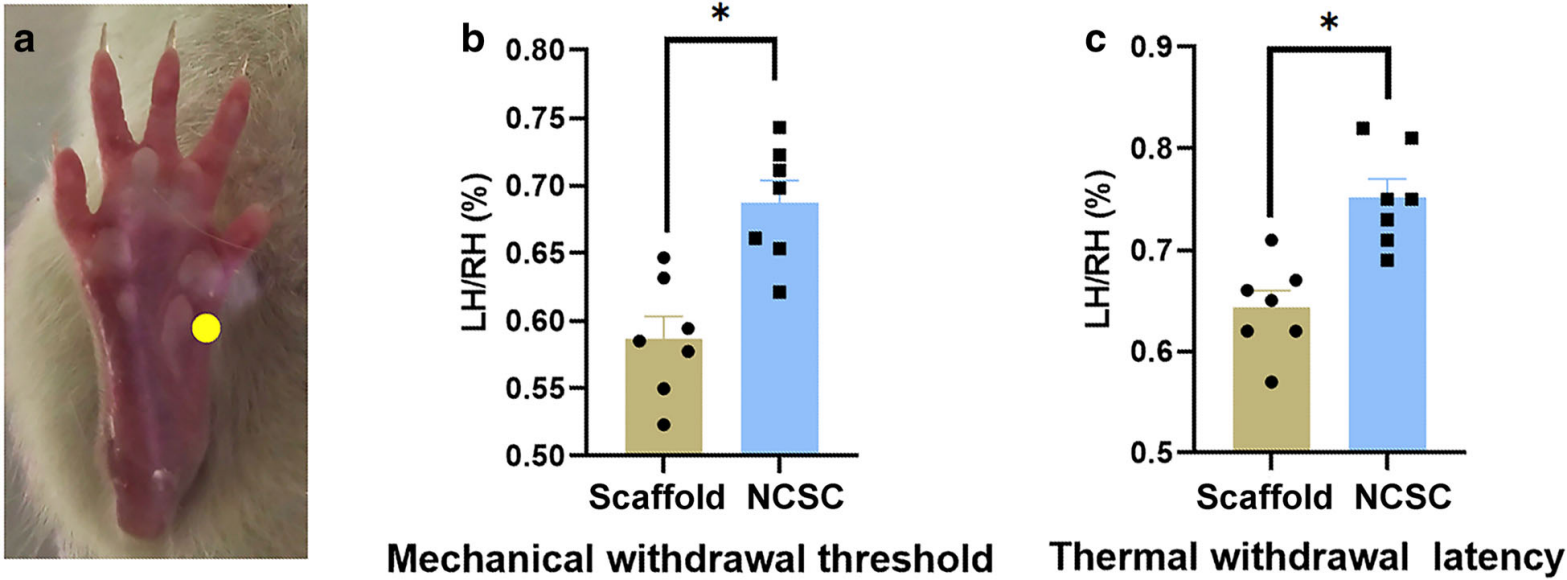
Sensory thresholds measured with algesimetry tests. **a** Assessment of sensory thresholds was conducted by applying mechanical (Von Frey test) and thermal (thermal plantar) stimuli to the medial site, corresponding to selective saphenous territories. More prevalent pain relief to SNT-induced mechanical allodynia (**b**) and thermal hyperalgesia (**c**) by NCSC treatment. *p < 0.05, *N* = 7

Mechanical and thermal allodynia in the medial-plantar surface of the ipsilateral hind paw 12 weeks after SNT were observed (Fig. 2a). SNT-induced pain was significantly relieved in the NCSC group (0.69 ± 0.02 for MWT; 0.75 ± 0.02 for TWL) when compared with the scaffold group (0.59 ± 0.02 for MWT; 0.64 ± 0.02 for TWL, both *p* < 0.05, Fig. 2b and c).

### Inhibition of glial activation

**Fig. 3:**
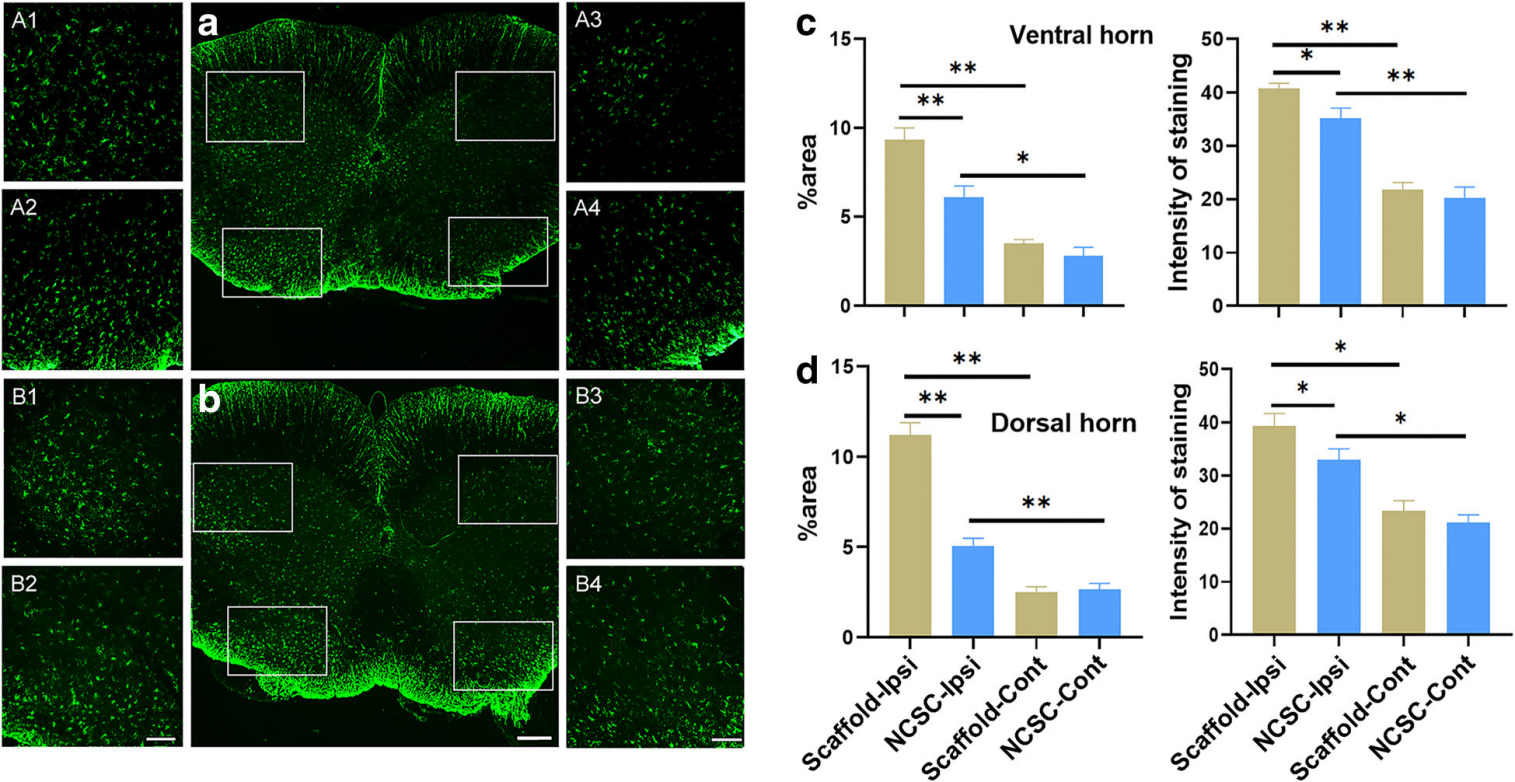
GFAP expression by immunofluorescence between two groups. The representative image of staining in the scaffold group (**a**) and the NCSC group (**b**). (A1–4) and (B1–4) are the respective magnification of the images in the white frame. (A1–2) and (B1–2) are ipsilateral side and (A3–4) and (B3–4) are contralateral side. Quantification of area percentage and averaged intensity of staining in the ventral (**c**) and dorsal horn (**d**). Astrocytes were activated after SNT, and the activation was significantly attenuated by NCSCs treatment. **a** and **b**, × 50, scale bar is 300 μm; (A1–A4) and (B1–B4), × 200, scale bar is 100 μm. *p < 0.05, **p < 0.01, *N* = 4

**Fig. 4:**
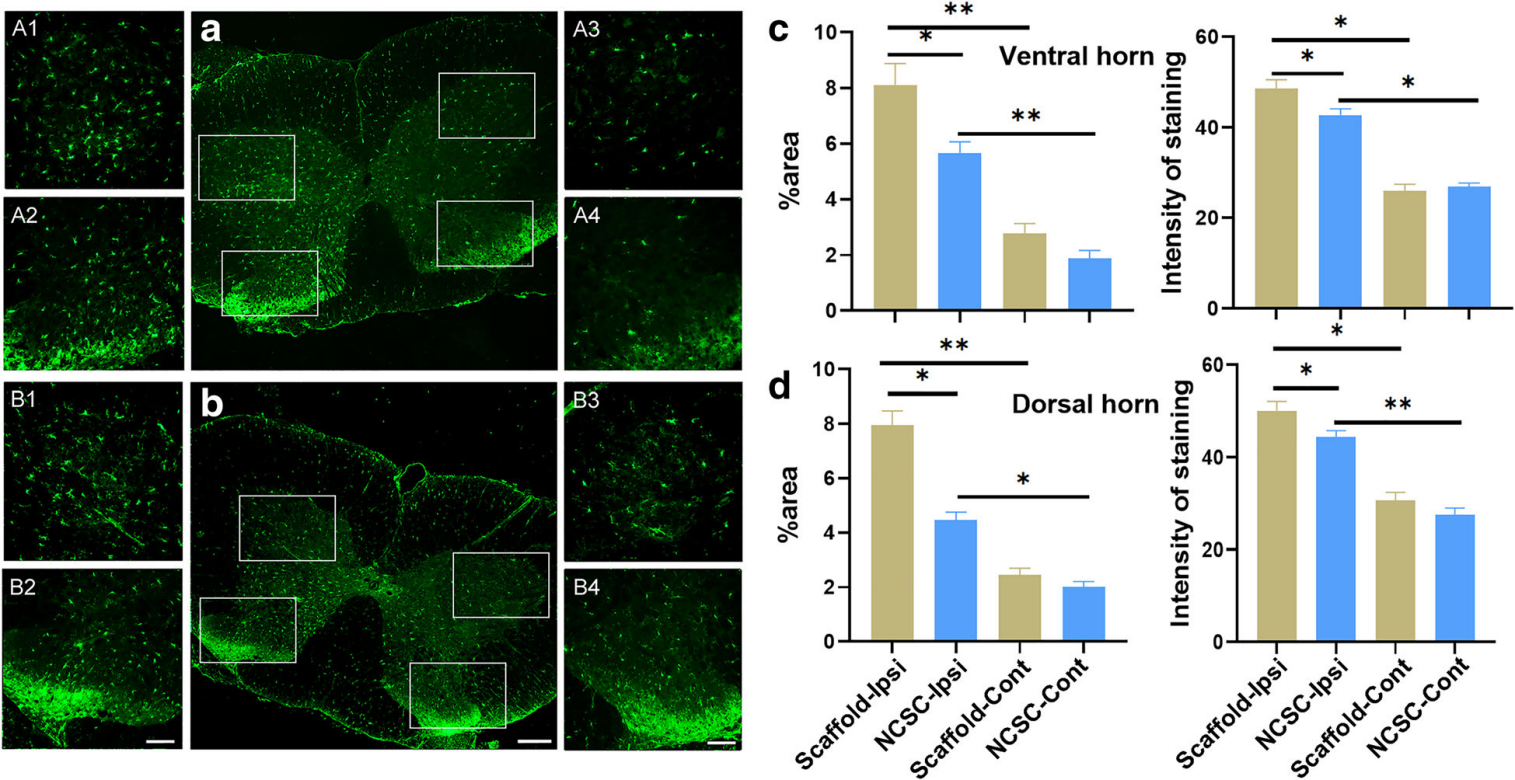
Iba-1 expression by immunofluorescence between two groups. The representative image of staining in the scaffold group (**a**) and the NCSC group (**b**). (A1–4) and (B1–4) are the respective magnification of the images in the white frame. (A1–2) and (B1–2) are the ipsilateral side and (A3–4) and (B3–4) are the contralateral side. Quantification of area percentage and averaged intensity of staining in the ventral (**c**) and dorsal horn (**d**). The microglia were activated after SNT and the activation were significantly attenuated by NCSCs treatment. **a** and **b**, × 50, scale bar is 300 μm; (A1–A4) and (B1–B4), × 200, scale bar is 100 μm. *p < 0.05, **p < 0.01, *N* = 4

SNT induced upregulation of the area percentage and the averaged intensity of GFAP (astrocyte) and Iba-1 (microglia) staining in the ipsilateral L4–5 dorsal and ventral horn compared with the contralateral side (Figs. 3 and 4, all *p* < 0.05). The area percentage of GFAP in the ipsilateral L4–5 ventral (6.11 ± 0.633 vs. 9.34 ± 0.66) and dorsal horn (5.07 ± 0.42 vs. 11.22 ± 0.69) was significantly attenuated by NCSC treatment when compared with scaffold group (both *p* < 0.01). The averaged intensity of GFAP staining in the ipsilateral L4–5 ventral (35.24 ± 1.87 vs. 40.75 ± 1.00) and dorsal horn (32.98 ± 2.09 vs. 39.40 ± 2.31) was also significantly attenuated by NCSC treatment when compared with the scaffold group (Fig. 3, both *p* < 0.05). The NCSC group showed lower area percentage and averaged Iba-1 staining intensity in the ipsilateral L4–5 ventral (5.67 ± 0.40 vs. 8.12 ± 0.76 for area percentage; 42.78 ± 1.42 vs. 48.67 ± 1.89 for averaged intensity, Fig. 4) and dorsal horn (4.46 ± 0.29 vs. 7.94 ± 0.54 for area percentage; 44.37 ± 1.43 vs. 50.14 ± 2.05 for averaged intensity). These results demonstrate that astrocyte and microglia activation were significantly attenuated by the NCSC treatment.

### Increased expression of neurotrophic factors

**Fig. 5:**
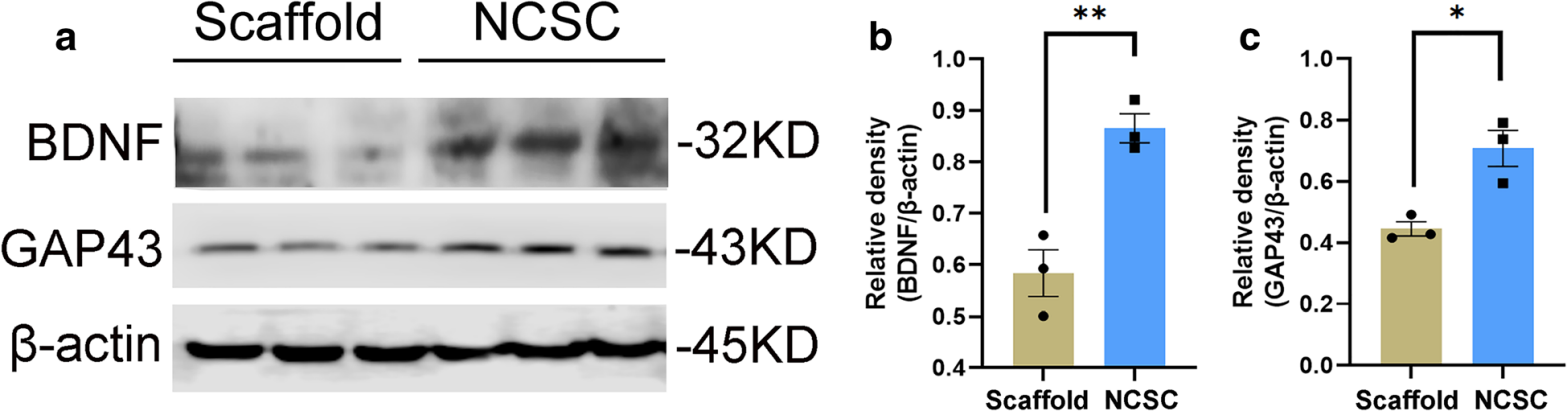
Expression of neurotrophic factors between two groups. Protein bands of BDNF and GAP-43 in the ipsilateral L4–5 spinal cord were detected by western blot analysis (**a**). Quantification of protein levels of BDNF (**b**) and GAP-43 (**c**). NCSC group had significantly higher neurotrophic factors expression compared with the Scaffold group. *p < 0.05, **p < 0.01, *N* = 3

The protein expression of neurotrophic factors in the NCSC group, including BDNF (0.87 ± 0.03 vs. 0.58 ± 0.05) and GAP-43 (0.71 ± 0.06 vs. 0.44 ± 0.02) in the ipsilateral L4–5 spinal cord, were significantly increased when compared with the scaffold group (both *p* < 0.05, Fig. 5).

### Decreased activation of ERK and NF-κB signals

**Fig. 6:**
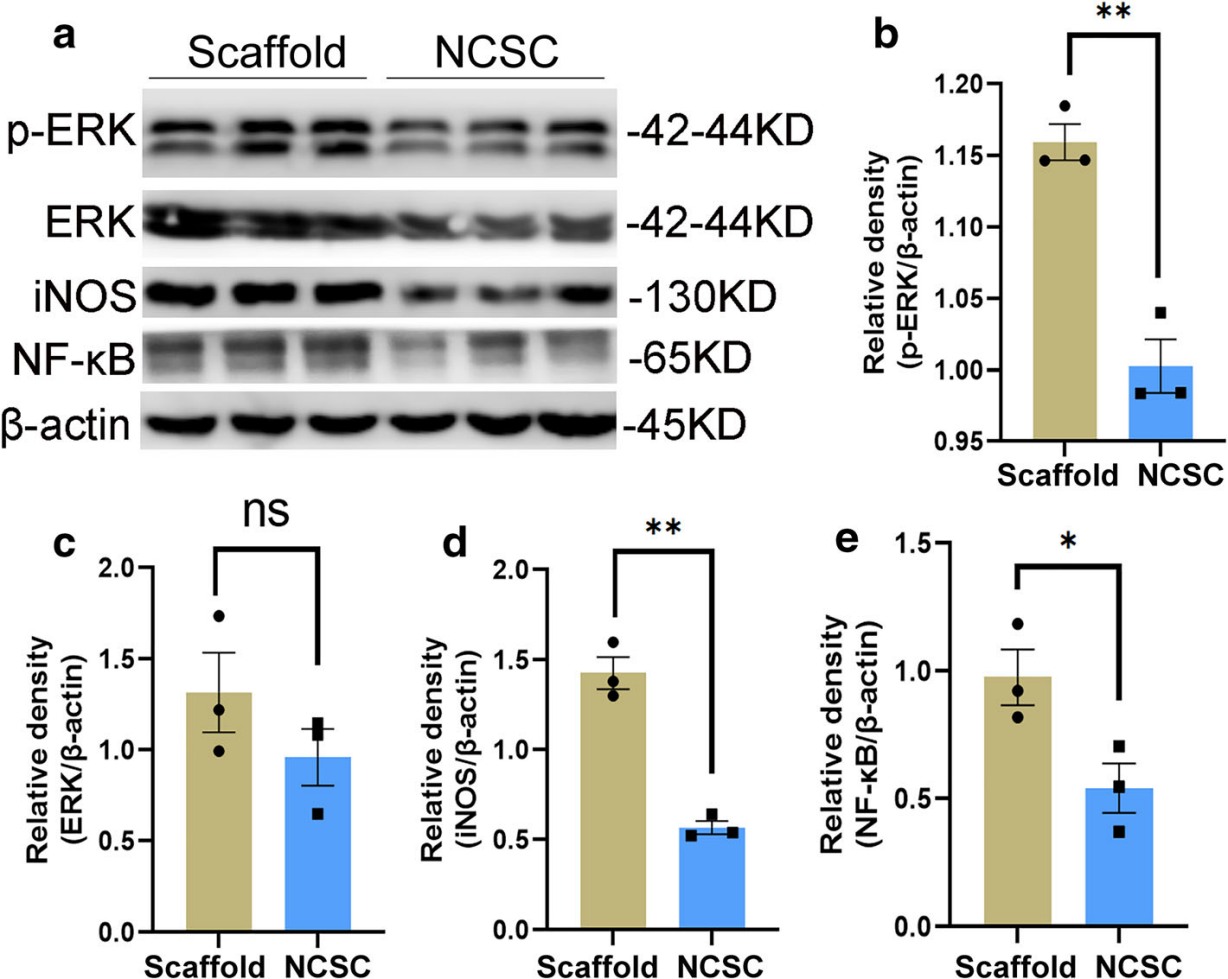
Protein bands of p-ERK, ERK, iNOS, and NF-κB p65 in the ipsilateral L4–5 spinal cord were detected by western blot analysis (**a**). Quantification of protein levels of p-ERK (**b**), ERK (**c**), iNOS (**d**), and NF-κB p65 (**e**). The decreased activation of ERK and NF-κB signals was observed in NCSC group. *p < 0.05, **p < 0.01, *N* = 3

The protein levels of p-ERK were significantly decreased in the NCSC group compared with the scaffold group (1.00 ± 0.02 vs. 1.16 ± 0.01, *p* < 0.05, Fig. 6b). While the expression of ERK exhibited a downward trend, there was not a statistically significant difference between the two groups (0.96 ± 0.16 vs. 1.32 ± 0.22, *p* = 0.26, Fig. 6c). The protein levels of iNOS (0.57 ± 0.04 vs. 1.43 ± 0.09) and NF-κB p65 (0.54 ± 0.10 vs. 0.98 ± 0.11) were also inhibited by NCSC treatment (both *p* < 0.05, Fig. 6d and e).

### Regulation of the expression of pain-related factors

**Fig. 7:**
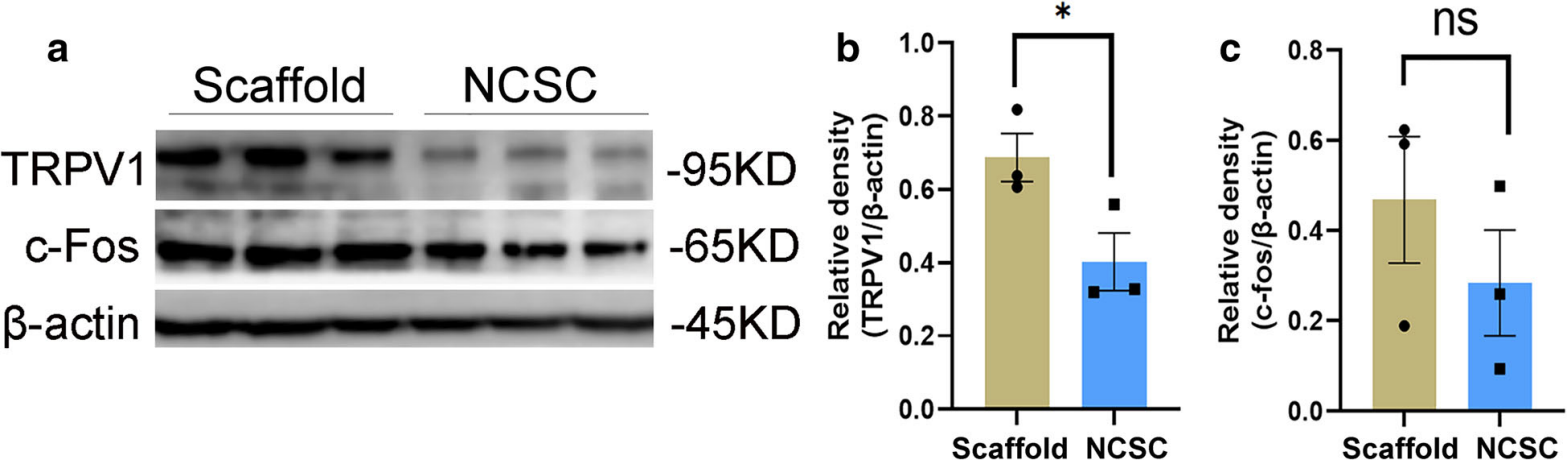
Protein bands of TRPV1 and c-fos in the ipsilateral L4–5 spinal cord were detected by western blot analysis (**a**). Quantification of protein levels of TRPV1 (**b**) and c-fos (**c**). The protein levels of TRPV1 were significantly decreased in NCSCs group, with no effect on c-fos expression. *p < 0.05, *N* = 3

The protein levels of TRPV1 were significantly decreased in the NCSC group compared with the scaffold group (0.40 ± 0.08 vs. 0.69 ± 0.07, *p* < 0.05, Fig. 7a and b). While the expression of c-fos, which is a marker of neural activation, exhibited a downward trend, there was no statistical difference between the two groups (0.28 ± 0.12 vs. 0.47 ± 0.14, *p* = 0.37, Fig. 7c). As shown in Supplementary Fig. 1, the intensity of CGRP staining was similar with the ipsilateral dorsal horn in both groups (36.38 ± 1.47 vs. 40.67 ± 2.21, *p* = 0.10), as well as similar with the contralateral side (35.94 ± 2.10 vs. 38.13 ± 1.72, *p* = 0.24).

## Discussion

**Fig. 8:**
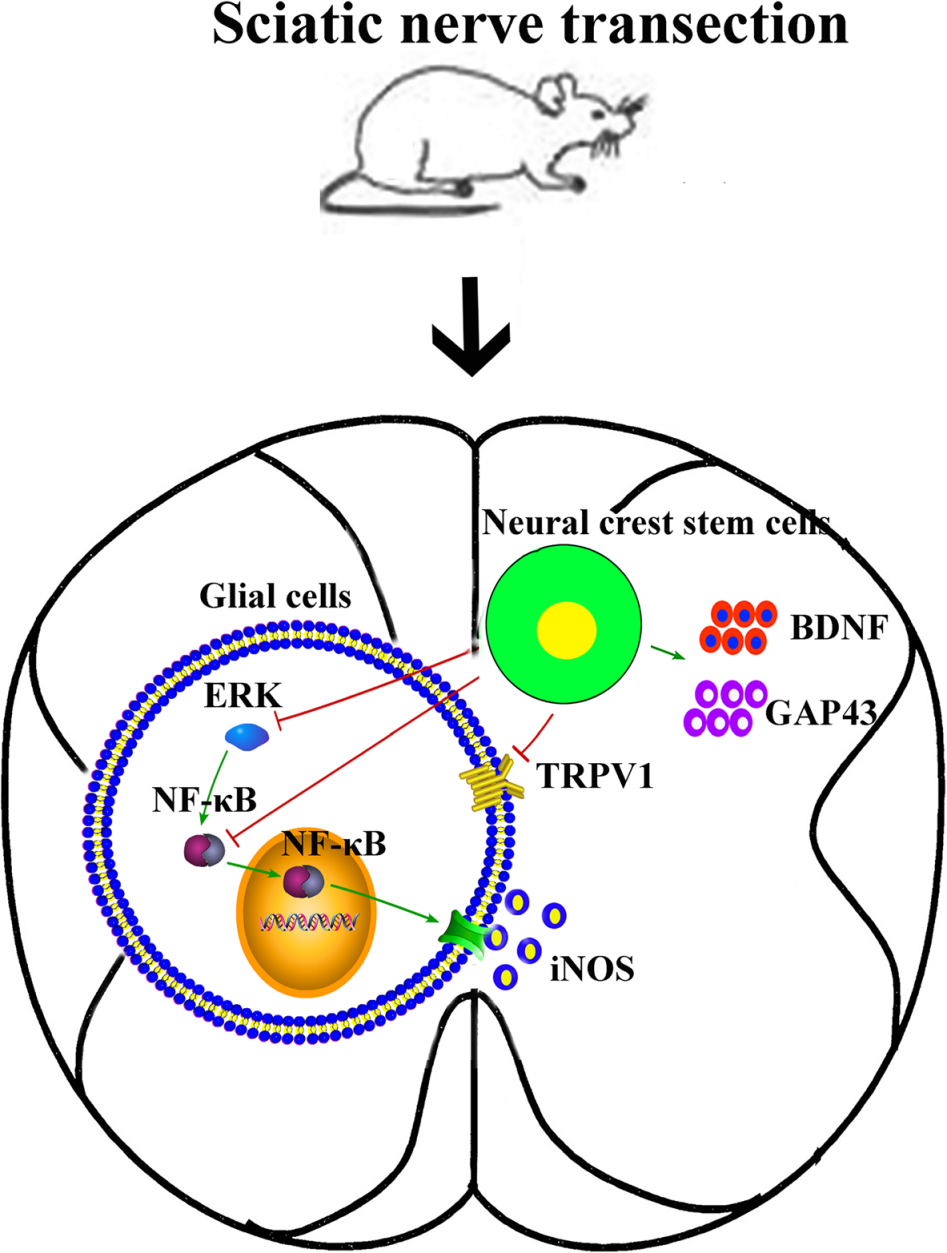
Proposed molecular mechanisms involved in therapeutic effects of 3D-printed scaffold-based NCSC therapy to the spinal cord after SNT. Our study suggested that the inhibition of ERK and NF-κB signaling pathways contributes to the decreased activation of glial and improvement of PWMT and TPWL. The increased expression of BDNF and GAP-43 may contribute to nerve recovery

In this study, we utilized a rat sciatic nerve defect model to demonstrate that NCSCs delivered at the injury site using a scaffold ameliorate neuropathic pain and enhance locomotion after PNI through inhibiting glial activation as well as ERK and NF-κB signals (schematically shown in Fig. 8). This novel peripherally administrated cell-based intervention facilitates central nerve regeneration and relieves central sensitization.

NCSCs, which can be isolated from embryos or produced from human pluripotent stem cells [33], are a transient and multipotent cell population that exhibit great potential for clinical tissue engineering and cell therapy applications[34]. NCSCs may provide trophic support to dorsal horn neurons to prevent them from degeneration [35, 36]or serve as a source for the substitution of damaged neurons [14]. Boundary cap neural crest stem cells (bNCSCs) implanted to the injured dorsal root transitional zone showed extensive survival and differentiated to neurons, forming elongated and apparently growth permissive tubes in the peripheral compartment of the dorsal root [37]. It was also shown that bNCSCs migrate into the spinal cord, express glial markers, and form elongated tubes, providing a conduit for regenerating sensory axons as well as a source for trophic support and cell replacement [14]. In previous studies, the location of NCSC transplantation was the dorsal root transitional zone, which is proximal to the spine [14, 37]. Here, we extend the therapeutic effects of NCSC by illustrating that the application of NCSCs to peripheral nerve can also provide protection to the spinal cord.

Both our prior study [23] and this study show that NCSC administration improves motor function. However, it is not clear whether NCSCs participate in the processes associated with neuropathic pain in the spinal cord after peripheral nerve injury. Our study showed that NCSCs could reverse SNT-induced mechanical and thermal hyperalgesia. The SNT model is a well-characterized model for sustained neuropathic pain and nerve regeneration [38–40]. Following sciatic nerve transection, all fibers of the tibial and peroneal nerves were cut to permanently disrupt the neurotrophic input to the soma. The mechanical and thermal hypersensitivity were attributed to in the intact saphenous nerve [41, 42]. A few days post lesion, the medial side of the paw showed marked hyperalgesia [39, 43]. The maintenance of neuropathic symptoms has been attributed to this altered pattern of reinnervation [44]. The hyperexcitability of the saphenous neurons could reflect chronic sensitization of its pathway, including spinal cord circuits [45]. It has been reported that the majority of the ectopic activity generated both after sciatic nerve and spinal segmental nerve injury is in A fibers [46]. Under different pathological conditions, such as central sensitization, the low threshold A-fiber afferents could “access” pain pathways [3, 47]. Therefore, our results suggest that NCSCs have an analgesic effect on neuropathic pain after SNT by the relief of central sensitization.

In order to elucidate the potential mechanisms associated with the prominent analgesic effect with motor function recovery obtained by the peripherally administrated NCSC therapy, we examined the changes in the spinal cord. First, the results demonstrated an increase in astrocyte and microglia activity on the ipsilateral side after SNT and that the NCGF group had a stronger inhibitory effect on the glial activation. It is reported that glial cell activation mediates long-term potentiation (LTP) induction in Aδ- and C-fibers, thus changing the neural excitability in the dorsal horn of the spinal cord in a spinal nerve ligation (SNL) model [48]. Meanwhile, activated microglia may phagocytize living neurons, resulting in neuronal death or loss [49]and neuropathic pain [50]. Moreover, our data revealed that the expression of growth-associated protein GAP-43 and BDNF were significantly increased on the affected side of the spinal cord in the NCSC group. The microenvironment of the injured spinal cord can influence the plasticity and regeneration capacity after nerve injury, such as the upregulation of inflammatory cytokines and excitatory neurotransmitters [51]. Thus, the transplantation of NCSCs in scaffolds could improve neuron survival and nociceptive relief. The therapeutic effects were inferred from the nutritional support and the suppression to the gliocyte activation. This synergic manner could facilitate spinal plasticity.

ERK and NF-κB p65 signaling pathways were activated after sciatic nerve injury, with increased protein expression, which are associated with spinal glial activation and central sensitization [25–27]. The double immunofluorescence labeling for c-fos and p-ERK was increased after the saphenous nerve injury and disconnection from their dominant receptive field, suggesting that the collateral synaptic input to second-order spinal dorsal horn neurons was activated [52]. ERK also mediates the release of a variety of pro-inflammatory mediators, such as TNF-α and NO from cultured microglial cells [53]. NF-κB/p65, a crucial transcription factor for maximal transcription of inflammatory molecules, is also involved in the modulation of neuropathic pain [54]. Translocation of NF-κB into the nucleus could trigger the activation of many cytokines, including IL-1β, IL-6, TNF-α, and iNOS [55]. Repeated intrathecal infusions of NF-κB inhibitor attenuated chronic constriction injury (CCI)-induced allodynia and hyperalgesia by inhibiting the activation of microglia and astrocytes [26]. According to reports, salidroside can reduce inflammatory response and promote motor function recovery in rats after spinal cord injury by inhibiting NF-κB, p38, and ERK signaling pathways [56]. Our results show that the expression of p-ERK, NF-κB, and iNOS was significantly decreased in the NCSC group, which was accompanied by significant pain relief. These results suggest that peripherally administrated NCSCs inhibit gliocyte activation by suppressing NF-κB and ERK signaling pathways.

TRPV1 is a nonselective cation channel in nociceptive sensory afferents, which mediates the release of neurotransmitters, such as glutamate and CGRP in the dorsal horn, and is a key factor involved in activating spinal glia in mice with nociceptive and pathological pain [57]. Furthermore, blockage of TRPV1 markedly promoted axonal regeneration following sciatic transection [58]. Inhibition of TRPV1 may also be one of the mechanisms associated with the therapeutic effect of NCSCs.

CGRP may contribute to the development and maintenance of peripheral sensitization and the associated hyperalgesia in inflammatory [59] and neuropathic pain [60]. After nerve crush, the expression of CGRP in the spinal cords increased. The immunoreactivity reached its peak at 11 days after lesion, followed by a gradual decline, and returned to normal levels 45 days after nerve crush [61]. C-fos may also play a crucial role in the development of the hyperalgesia [62]. In our research, there was no difference between the scaffold and NCSC groups 12 weeks after SNT, suggesting the CGRP and c-fos may not participate in the neuropathic pain at this stage.

Directly grafting stem cells to the spinal cord has been shown to exert plastic changes by reconstructing neural circuits, providing trophic supports, and inhibiting injury-induced inflammatory response [51]. While the spinal cord was not the primary injury area after peripheral injury, compared to the status after spinal cord injury, the synapses and neural circuits in the spinal cord have also been significantly damaged. The inhibited ERK and NF-κB signaling pathways reduced glial cell activation and increased NGF expression, suggesting that NCSCs indirectly contribute to the spinal plasticity. While our work suggested ERK and NF-κB signal pathways are involved, there could also be additional contributing pathways. Such mechanism-focused studies are an important aspect of understanding the therapeutic efficacy of NSCSs and should be considered in future research. The abovementioned signaling pathways would be verified in cells by using relevant inhibitors or adenoviruses in future research to have a deeper understanding the therapeutic efficacy of NSCSs.

In conclusion, NCSC-laden scaffolds can reverse neuropathic pain associated with SNT and improve motor function recovery. Protein expression analyses suggest that the mechanisms underlying this treatment could be associated with ERK and NF-κB signal downregulation and glia cell suppression, which contribute to spinal plasticity. This work also supports the use of peripheral NCSC transplantation as a valuable intervention for neuropathic pain and nerve regeneration after PNI.

## Supplementary Information

**Additional file 1:** **Supplementary Fig. 1.** Images of stained CGRP expression detected by immunofluorescence among the two groups. The representative image of staining and the magnification of the image in the Scaffold group (A) and NCSC group (B). Quantification of averaged intensity of staining in the dorsal horn (C). The intensity of staining of CGRP was similar with the ipsilateral dorsal horn of these two groups, as well as similar with the contralateral side. A and B: × 50, scale bar is 300 μm; The magnification of the image: × 200, scale bar is 100 μm. *p < 0.05, **p < 0.01, *N* = 4

## Data Availability

Not applicable.

## Abbreviations

NCSC: Neural crest stem cells
SNT: Sciatic nerve transection
PWMT: Paw withdrawal mechanical threshold
TPWL: Thermal paw withdrawal latency
BDNF: Brain-derived neurotrophic factor
GAP: Growth-associated protein
CGRP: Calcitonin gene-related peptide
PNI: Peripheral nerve injury
BMSCs: Bone marrow mesenchymal stem cells
NCSC: Neural crest stem cells
TRPV1: Transient receptor potential vanilloid 1
i-NOS: Inducible nitric oxide synthase
GFAP: Glial fibrillary acidic protein
Iba-1: Ionized calcium-binding adapter molecule 1
bNCSCs: Boundary cap neural crest stem cells
SNL: Spinal nerve ligation
CCI: Chronic constriction injury
