# Supplementary figures and images for: Therapeutic effects of peripherally administrated neural crest stem cells on pain and spinal cord changes after sciatic nerve transection
Source: Stem Cell Res Ther. 2021 Mar 15;12:180. doi: 10.1186/s13287-021-02200-4 (PMC7962265; doi:10.1186/s13287-021-02200-4)

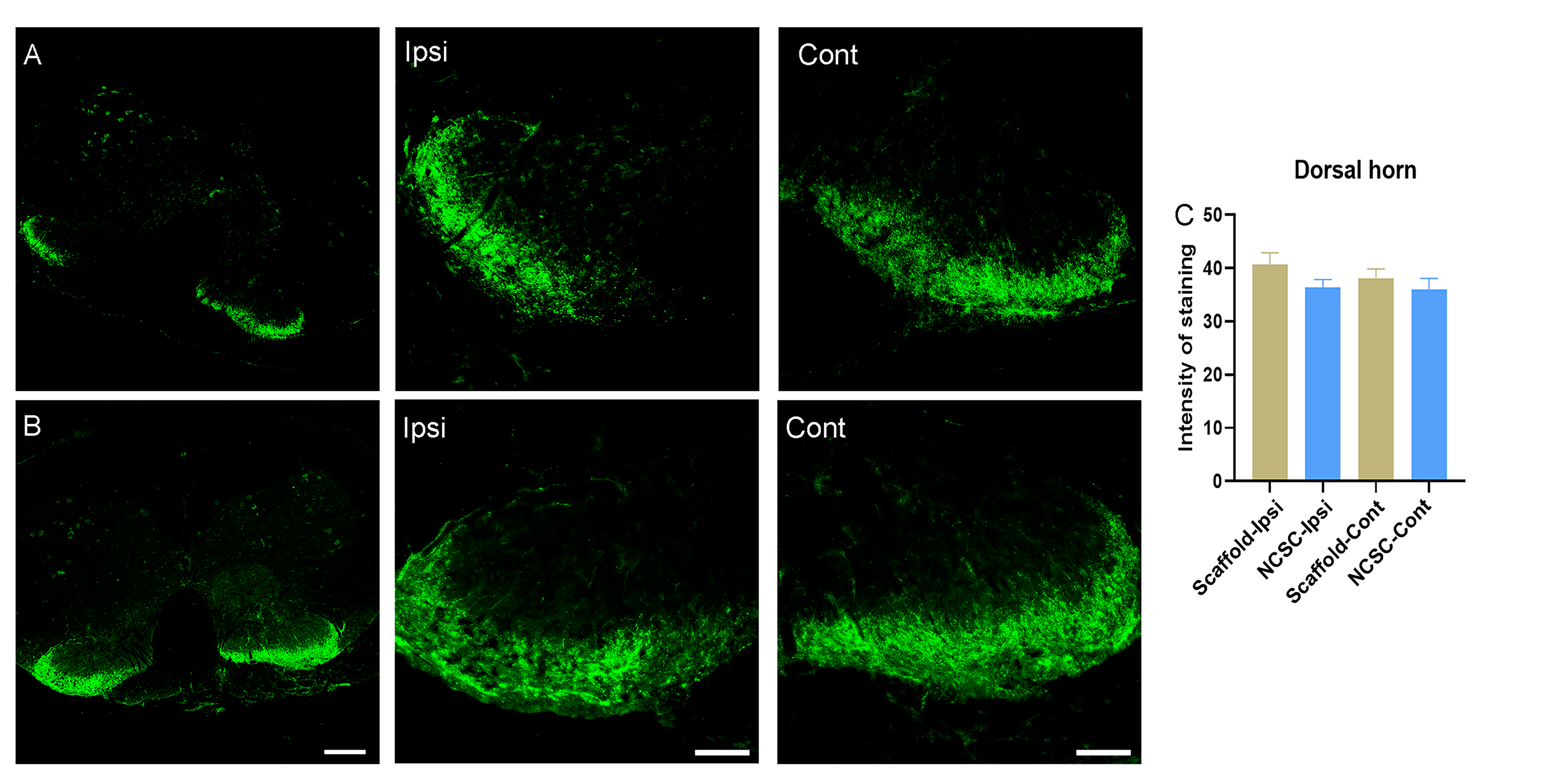

Supplement: Supplementary file 1 — Additional file 1: Supplementary Fig. 1. Images of stained CGRP expression detected by immunofluorescence among the two groups. The representative image of staining and the magnification of the image in the Scaffold group (A) and NCSC group (B). Quantification of averaged intensity of staining in the dorsal horn (C). The intensity of staining of CGRP was similar with the ipsilateral dorsal horn of these two groups, as well as similar with the contralateral side. A and B: × 50, scale bar is 300 μm; The magnification of the image: × 200, scale bar is 100 μm. *p < 0.05, **p < 0.01, N = 4 [file 13287_2021_2200_MOESM1_ESM.tif]
